# FFA2 Contribution to Gestational Glucose Tolerance Is Not Disrupted by Antibiotics

**DOI:** 10.1371/journal.pone.0167837

**Published:** 2016-12-13

**Authors:** Miles Fuller, Xiaoran Li, Robert Fisch, Moneb Bughara, Barton Wicksteed, Petia Kovatcheva-Datchary, Brian T. Layden

**Affiliations:** 1 Division of Endocrinology, Metabolism and Molecular Medicine, Northwestern University Feinberg School of Medicine, Chicago, Illinois, United States of America; 2 Wallenberg Laboratory, Department of Molecular and Clinical Medicine, University of Gothenburg, Gothenburg, Sweden; 3 Jesse Brown Veterans Affairs Medical Center, Chicago, Illinois, United States of America; 4 Division of Endocrinology, Diabetes, and Metabolism, Department of Medicine, University of Illinois at Chicago, Chicago, Illinois, United States of America; Universidade do Estado do Rio de Janeiro, BRAZIL

## Abstract

During the insulin resistant phase of pregnancy, the mRNA expression of free fatty acid 2 receptor (*Ffar2*) is upregulated and as we recently reported, this receptor contributes to insulin secretion and pancreatic beta cell mass expansion in order to maintain normal glucose homeostasis during pregnancy. As impaired gestational glucose levels can affect metabolic health of offspring, we aimed to explore the role of maternal *Ffar2* expression during pregnancy on the metabolic health of offspring and also the effects of antibiotics, which have been shown to disrupt gut microbiota fermentative activity (the source of the FFA2 ligands) on gestational glucose homeostasis. We found that maternal *Ffar2* expression and impaired glucose tolerance during pregnancy had no effect on the growth rates, *ad lib* glucose and glucose tolerance in the offspring between 3 and 6 weeks of age. To disrupt short chain fatty acid production, we chronically treated WT mice and *Ffar2*^*-/-*^ mice with broad range antibiotics and further compared their glucose tolerance prior to pregnancy and at gestational day 15, and also quantified cecum and plasma SCFAs. We found that during pregnancy antibiotic treatment reduced the levels of SCFAs in the cecum of the mice, but resulted in elevated levels of plasma SCFAs and altered concentrations of individual SCFAs. Along with these changes, gestational glucose tolerance in WT mice, but not *Ffar2*^*-/-*^ mice improved while on antibiotics. Additional data showed that gestational glucose tolerance worsened in *Ffar2*^*-/-*^ mice during a second pregnancy. Together, these results indicate that antibiotic treatment alone is inadequate to deplete plasma SCFA concentrations, and that modulation of gut microbiota by antibiotics does not disrupt the contribution of FFA2 to gestational glucose tolerance.

## Introduction

The gut microbiota is now well described to influence different metabolic states [1,2]. Of importance to this study, the human gut microbiota has been shown to undergo dynamic changes during pregnancy and drive metabolic changes in the host [3]. However, the mechanisms by which the gut microbiota mediate metabolic change including during pregnancy remain unknown. A g-protein-coupled receptor (GPCR), free fatty acid 2 receptor (FFA2; GPR43), expressed in pancreatic beta (β) cells contributes to glucose stimulated insulin secretion (GSIS) [4,5], and *Ffar2* levels in islets are elevated during pregnancy suggesting a potential role for FFA2 in the β cell response to gestational insulin resistance [6,7]. The ligands for FFA2, short chain fatty acids (SCFAs), are the products of fermentation by the gut microbiota [8,9]. These data suggest that FFA2 may be mediating gestational glucose homeostasis and this function may be regulated by changes in the gut microbiota.

Due to this relationship, our group recently investigated the role of FFA2 in gestational glucose homeostasis using genetic FFA2 global knockout (KO; *Ffar2*^-/-^; *Gpr43*^-/-^) mice. We showed that at the peak of gestational insulin resistance in mice, gestational day 15 (G15) [10], FFA2 is necessary to maintain glucose tolerance, glucose stimulated insulin secretion and β cell mass expansion [11]. We also observed that at phylum level the gut microbiota composition is influenced by pregnancy with consequential changes in cecum and blood SCFA levels [11]. These data suggested a relationship between FFA2, gut microbiota and SCFAs during pregnancy that regulates gestational glucose homeostasis. These above published findings have raised multiple important avenues of investigation, two of which are explored here.

Maternal hyperglycemia during pregnancy is associated with abnormally high insulin resistance, fetal hyperinsulinemia and overgrowth [12]. These changes, which are associated with the diagnosis of gestational diabetes, complicate pregnancies by increasing the occurrence of hypertensive disorders, necessity for cesarean section, presence of macrosomia in newborns, risk of shoulder dystocia and prenatal mortality. In the long-term, it increases the likelihood that the mother will develop type 2 diabetes mellitus (T2DM), cardiovascular disease (CVD), and metabolic syndrome [13]. Similarly, maternal hyperglycemia during pregnancy places the offspring at increased risk for T2DM, obesity and metabolic syndrome [14]. Therefore, we first explore if maternal hyperglycemia during pregnancy due to *Ffar2* deletion is sufficient to lead to aberrant metabolic health in their offspring.

Our earlier report [11] suggested that the gut microbiota-SCFAs-FFA2 relationship is necessary to maintain maternal glucose homeostasis where absence of the sensor, FFA2, was responsible for impaired gestational glucose in mice. This raises the possibility that a void in stimuli (SCFAs) or stimuli producers (gut microbiota) can modulate the contribution of FFA2 to glucose homeostasis. Several methods exist to study the contribution of gut microbiota to host functions. For example, gnotobiotic mice are used to determine the impact of specific microbial cohorts [15]. While gnotobiotic mice allow for the highly controlled study of gene function in the presence of known gut microbes, antibiotic ablation is an alternative approach to evaluate the impact of the gut microbiota on host health [16] that can be easily applied. Studies have shown that chronic, broad-range antibiotic treatment effectively depletes the gut microbial population in mice and such antibiotic knockdown has been used to explore effects of the gut microbiota on various metabolic parameters [17]. Considering that FFA2 is required for normal gestational glucose homeostasis and activated by gut-derived metabolites, we also examined the role of the gut microbiota in gestational glucose homeostasis through antibiotic knockdown of the gut microbiota.

Therefore, here, we conducted two studies: 1) an *offspring study*, where the metabolic health of progeny from WT and *Ffar2*^-/-^ mothers with or without impaired gestational glucose tolerance was evaluated and 2) an *adult study*, in which the glucose tolerance of mice were evaluated at G15 after antibiotic knockdown of the gut microbiota. This study aims to provide new insights into how disruption of the gut microbiota-SCFAs-FFA2 relationship affects glucose homeostasis in mothers and determine if this relationship impacts the metabolic health of the offspring.

## Methods

### Animals

*Ffar2*^+/-^ mice were bred to generate *Ffar2*^-/-^ mice and wild-type (WT) mice as previously described [18]. In brief, the *Ffar2* has 3 exons, a portion of exon 1 was replaced by a targeting sequence resulting in a frame shift mutation of the downstream amino acid sequence. Genotype was determined by PCR, where genomic DNA was amplified by PCR using multiplex primer pairs as before [11]. Male and female mice were studied between 3 and 25 weeks of age as indicated in the results section. All animal experiments were approved by the Institutional Animal Care and Use Committee at Northwestern University. For the following experiments, mice were euthanized with CO_2_ exposure followed by decapitation as confirmation. Newborns were considered to die after birth if discovered in the first 1–2 days following observing a new litter. All mice were on a standard chow diet (3.1 Kcal/g) from LM-485 Harlan laboratories, which includes % kcal from protein (25%), carbohydrate (58%), and fat (17%).

### Breeding approach for Offspring Study

**Fig 1:**
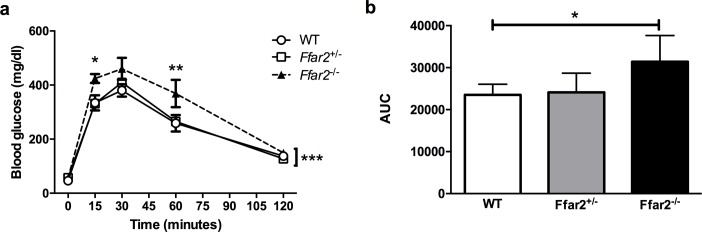
*Ffar2*^+/-^ mice exhibit normal glucose tolerance at gestational day 15 (G15). (**a**) Plasma glucose concentrations from WT, *Ffar2*^+/-^ and *Ffar2*^-/-^ female mice during an IPGTT at G15. (**b**) The corresponding area under the curve (AUC) for the IPGTT. WT circles and white bars; *Ffar2*^+/-^, squares and gray bars; *Ffar2*^-/-^, triangles and black bars. Data in (a) were compared by 2-way ANOVA with Bonferroni post-hoc analyses. Data in (b) were compared by Student’s t-test (*, p<0.05; **, p<0.01; ***p<0.001), n = 4–5 mice/ group.

**Fig 2:**
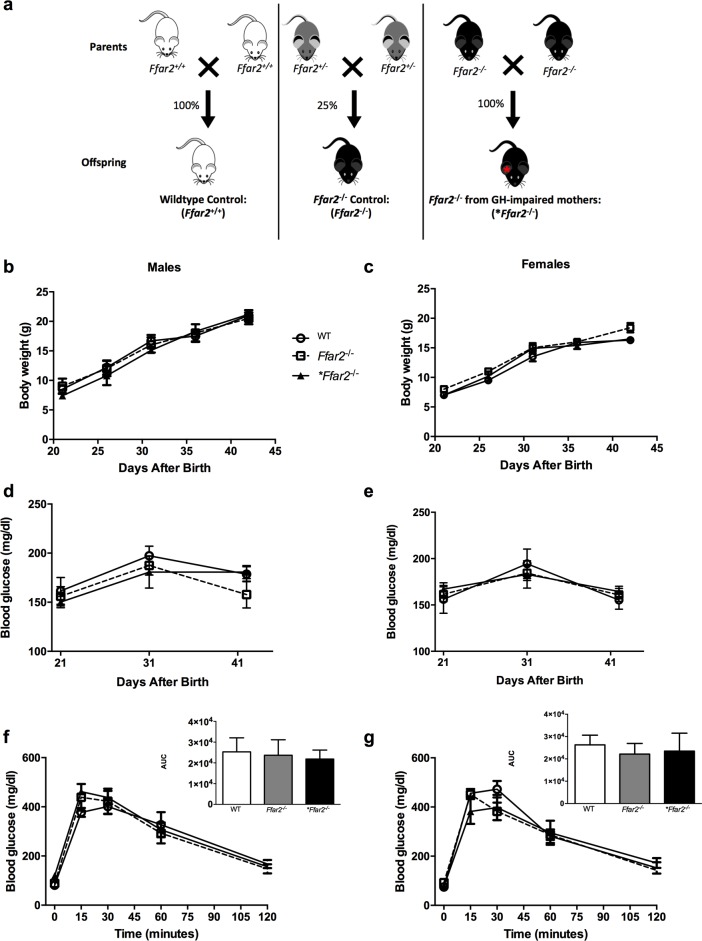
*WT*, *Ffar2*^-/-^ and *Ffar2^-/-^ mice exhibit similar weight gain, random glucose levels and glucose tolerance at 6 weeks of age. (**a**) Breeding scheme to generate these offspring is shown. For **b-g**, changes in body weight (**b-c**), *ad lib* plasma glucose concentrations (**d-e**) and plasma glucose concentrations during an IPGTT (**f-g**) with male (**b, d** and **f**) and female (**c, e** and **g**) *WT*, *Ffar2*^-/-^ and *Ffar2^-/-^ offspring are shown. Inserts for **f** and **g** represent the area under the curve for the IPGTT. WT, circles and white bars; *Ffar2*^-/-^, squares and gray bars; *Ffar2^-/-^, triangles and black bars. Data are represented as mean ± SEM (*p ≤ 0.05), n = 3–12. Data in (b-g) were compared by 2-way ANOVA with Bonferroni post-hoc analyses.

For the offspring study, WT x WT, *Ffar2*^+/-^ x *Ffar2*^+/-^ and *Ffar2*^-/-^ x *Ffar2*^-/-^ breeding pairs were used and had glucose tolerance evaluated (see **Fig 1**). The offspring included two control groups; WT mice and *Ffar2*^*-*/-^, and the experimental *Ffar2^-/-^ group, respectively, generated from these breeding pairs (see **Fig 2A**). From these same breeding pairs, the offspring were followed and metabolically assessed, as reported in **Fig 2**and **Table 1**.

**Table 1:** Litter size and morbidity rate

Breeding Group	Newborn No.		Newborns That Died After Birth	
	Total no.	No./litter	Total no.	No./litter
*Ffar2*^*+/+*^ *x Ffar2*^*+/+*^	36	5.1 ± 0.9	2	0.15 ± 0.08
*Ffar2*^*+/-*^ *x Ffar2*^*+/-*^	32	5.6 ± 1.1	2	0.08 ± 0.06
*Ffar2*^*-/-*^ *x Ffar2*^*-/-*^	31	6.2 ± 1.0	1	0.04 ± 0.04

Values are means ± SE.

### Antibiotic Treatment for Adult Study

At 8 weeks of age, mice were given water supplemented with antibiotics (1g/L of neomycin, ampicillin and metronidazole; Sigma) [17]. Antibiotic solutions were made fresh and replaced every 3 days. After 3 weeks of antibiotic treatment, WT and *Ffar2*^-/-^ females were mated with *Ffar2*^+/-^ males. Following copulation, the male breeders were removed and the experimental mice were housed individually. Upon delivery, the experimental mice were taken off the antibiotic treatment and their microbiota was reconstituted with 50μL of the fecal suspension via oral gavage. To generate the fecal suspension, ~3g of fresh fecal pellets were collected from the *Ffar2*^+/-^ breeding pairs used to generate the experimental mice, homogenized and suspended in 10mL of PBS, aliquoted in 200μL and were stored at -80°C until ready for use. Two weeks after reconstitution, the experimental females were mated with an antibiotic-treated male breeder. Breeders were once again removed following copulation and pregnant females were housed individually. Plasma from blood, fecal and cecal samples were collected at the defined time points and stored at -80°C until further use.

### Glucose and Insulin Tolerance Tests

Intraperitoneal glucose tolerance tests (IPGTTs) were done on mice fasted overnight with glucose given by intraperitoneal injection (2g glucose/kg of body weight). Blood was obtained from tail veins for glucose determination (measured with a One-Touch Ultra Glucometer). Glucose levels were measured at multiple time points (from 0 to 120 min) during the IPGTT. For the IPGTT, area under the curve (AUC) values were calculated by standard approaches using the trapezoidal rule and are represented as mean ± SEM. Blood obtained from tail veins at 0, 5 and 15 mins during the IPGTT was used to measure insulin levels by ELISA assay (ALPCO). Insulin tolerance tests (ITT) were performed on mice fasted for 6 hrs, by intraperitoneal injections of 1.0 U/kg insulin and glucose levels followed over 120 min. Results are presented as percentage of blood glucose before insulin injection.

### Organic Acids Extraction and Analysis

Gas chromatography-mass spectrometry (GC-MS) was used for measurement of organic acids in mouse plasma collected from inferior vena cava and cecal samples as fully described in Fuller *et al*. [11]. Both the cecum and plasma samples were processed and extracted including the addition of internal standards as before [11], and an aliquot of the resulting derivatized material was injected into a gas chromatograph (Agilent Technologies 7890 A) coupled to a mass spectrometer detector (Agilent Technologies 5975 C) for analysis. For the cecal analysis, both SCFAs (acetate, propionate, and butyrate), and the SCFA precursors (lactate and succinate) were measured and reported. For the plasma metabolites, only the SCFAs (acetate, propionate, butyrate) were measured and reported.

### GLP-1 Measurements

For measuring glucagon-like peptide 1 (GLP-1) secretion, 2 g glucose/kg of body wt OGTT was performed with blood samples collected at 0 and 30 min using heparin capillary tubes (Drummond, Broomall, PA) and stored in microcentrifuge tubes containing 50μM DPP-IV inhibitor (EMD Millipore, St. Charles, MO). Samples were then centrifuged for 10 minutes at 7,000 x g and plasma separated for storage at -80°C until analysis [11]. The ELISA kit for active GLP-1 was from Immuno-Biological Laboratories (Minneapolis, MN).

### Bacterial 16S DNA Expression

DNA was isolated from fecal samples using the PowerSoil kit (MoBIO) according to Earth Microbiome Project (EMP) standard protocols (http://www.earthmicrobiome.org/emp-standard-protocols/dna-extraction-protocol/) and quantified by spectrophotometry at 260 nm. Primers for V2 and V6 region of bacterial 16S rRNA genes and mouse genomic DNA were used as previously described [19]. Each 20μl PCR reaction contained 7μl of MoBio PCR Water (Certified DNA Free), 10μl PerfeCTa SYBR Green FastMix (2X), 1μl of Forward Primer (0.25μM final concentration), 1μl of Reverse Primer (0.25μM final concentration), and 1μl DNA template (0.0625ng DNA for 16S V2 and V6, 25ng DNA for mouse genomic DNA). The PCR parameters were as follows: 3min activation step (95°C); then 40 cycles of 3 s denaturation (95°C), 30 s annealing and extension (60°C for 16S-V2 and mouse genomic, 55°C for 16S-V6). For each sample, the number of 16S DNA copies was related to the number of mouse genomic DNA copies. Samples with a threshold more than 35 cycles for the genomic PCR were regarded as poorly amplifiable and excluded from analysis.

### Statistical Analysis

Values are reported as the mean ± SEM. *P* values were calculated by Student’s *t*-test (two-tailed) and 2-way ANOVA with Bonferroni post-hoc analyses. *P* < 0.05 was considered significant.

## Results

### Impaired glucose tolerance in *Ffar2*^-/-^ mice does not lead to acute metabolic effects on their offspring

Previously, we observed that female *Ffar2*^-/-^ mice exhibit impaired glucose tolerance during pregnancy, at a time of heightened insulin resistance, as compared to control mice [11]. Since gestational diabetes mellitus (GDM) in humans significantly increases the likelihood of adverse effects on the offspring’s metabolic health [1], we assessed whether the glucose tolerance impairment in the *Ffar2*^-/-^ mothers impacted the glucose homeostasis of their offspring. To evaluate this question, a comparison in metabolic parameters of offspring from the following breeders (WT x WT, *Ffar2*^+/-^ x *Ffar2*^+/-^ and *Ffar2*^-/-^ x *Ffar2*^-/-^) was done, specifically comparing both female and male WT, *Ffar2*^-/-^ and *Ffar2^-/-^ offspring from each of these breeding pairs, respectively. This approach allowed us to examine the influence of genotype (WT versus *Ffar2*^-/-^) as well as maternal glucose tolerance on the offspring (see **Fig 2A**). Therefore, we first determined if glucose tolerance was altered in *Ffar2*^+/-^ as compared to WT and *Ffar2*^-/-^ mothers during pregnancy (at G15). Confirming our previous results, *Ffar2*^-/-^ mice had significantly impaired glucose tolerance relative to WT mice during pregnancy (**Fig 1A and 1B**) at G15, and *Ffar2*^+/-^ female mice had similar glucose tolerance to the WT mice during pregnancy. These later data suggest that one copy of the *Ffar2* gene is sufficient to maintain normal gestational glucose tolerance in mice. Because the *Ffar2*^+/-^ female mice do not develop impaired glucose tolerance, this allows us to assess the influence of impaired maternal glucose tolerance on the metabolic health of the *Ffar2*^-/-^ offspring.

To determine the impact of impaired glucose tolerance on the early life of offspring in this model, we further bred WT x WT, *Ffar2*^+/-^ x *Ffar2*^+/-^ and *Ffar2*^-/-^ x *Ffar2*^-/-^ breeding pairs and we compared the litter size and survival rates of their offspring (**Table 1**). The mean litter sizes and stillbirth rates were similar across each of the breeding pairs (**Table 1**). These results suggest that maternal impairment in glucose tolerance due to FFA2 deletion does not influence litter size or survival. To investigate whether impairment in maternal glucose tolerance affects growth of the progeny, we examined both female and male WT offspring from WT x WT pairs and *Ffar2*^-/-^ offspring from *Ffar2*^+/-^ x *Ffar2*^+/-^ and *Ffar2*^-/-^ x *Ffar2*^-/-^ breeding pairs (**Fig 2A**). As apparent, female and male KO mice (*Ffar2^-/-^) from *Ffar2*^-/-^ x *Ffar2*^-/-^ breeding pairs that were exposed to impairment in maternal glucose tolerance had similar body weights from the weaning date (21 days after birth) to sexual maturity (42 days after birth) as compared to KO offspring (*Ffar2*^-/-^) from *Ffar2*^+/-^ x *Ffar2*^+/-^ and WT offspring from WT x WT breeding pairs (**Fig 2B and 2C**). Thus, impaired maternal glucose tolerance in the *Ffar2*^-/-^ mice does not influence offspring body weight or growth during this period.

As weight or growth may not be the only parameters influenced by altered maternal glucose tolerance, we next evaluated its effects on glucose homeostasis in the offspring by measuring blood glucose *ad libitum* and during an IPGTT (at 6 weeks of age). When comparing WT offspring from WT x WT pairs and *Ffar2*^-/-^ offspring from *Ffar2*^+/-^ x *Ffar2*^+/-^ and *Ffar2*^-/-^ x *Ffar2*^-/-^ breeding pairs, *ad libitum* blood glucose measurements was similar in male and female offspring between days 21 and 42 across each group (**Fig 2D and 2E**). IPGTTs were conducted at 42 days of age to more rigorously assess glucose tolerance and showed no significant difference in the blood glucose levels between groups in either sex (**Fig 2F and 2G**). These results verify that *Ffar2* deletion impairs maternal glucose tolerance, but this impairment does not affect the growth, random glucose levels or glucose tolerance of the offspring in the first 6 weeks of life.

### Antibiotic disruption of the gut microbiota leads to improved glucose tolerance independent of FFA2

**Fig 3:**
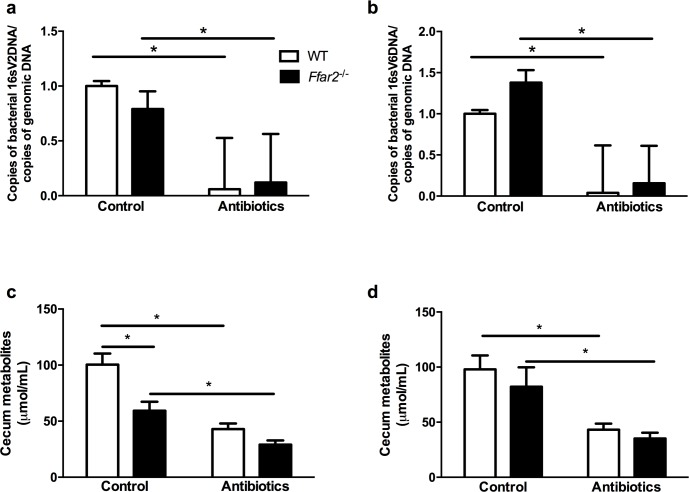
Antibiotic treatment significantly reduces cecum metabolites concentrations. Antibiotic treatment significantly reduced cecum bacterial populations for the V2 regions (**a**) and V6 regions (**b**) with both WT and *Ffar2*^-/-^ mice, where the copies of 16S V2 and V6 DNA copies (which reflects the amount of bacteria) was relative to the copies of mouse genomic DNA. Total metabolite levels, which includes both the SCFAs (acetate, propionate, and butyrate) and intermediates in SCFA synthesis (succinate and lactate) in cecum samples are shown from *Ffar2*^-/-^ and WT mice before pregnancy (**c**) and at G15 (**d**) under control and antibiotic-treated conditions. WT, white bars; *Ffar2*^-/-^, black bars. Data are represented as mean ± SEM (*p ≤ 0.05), n = 4–8 for **a-b**, n = 6–8 for **c-d**. Data (for **a-d**) were compared by Student’s t-test. (*, p<0.05).

Our lab recently described a link between pregnancy-associated changes in the gut microbiota, plasma SCFA levels and β cell specific *Ffar2* expression [11] in the maintenance of gestational glucose homeostasis. Since high-dose antibiotic treatment has been shown to significantly deplete the gut microbial load [17], we tested whether antibiotic knockdown of the gut microbiota could disrupt this novel relationship. Thus, we first assessed if our approach, of giving drinking water supplemented with antibiotics, successfully knocked down the gut microbiota, where we observed that WT and *Ffar2*^*-/-*^ mice had dramatically depleted the bacterial population of the gut microbiota by 16S DNA expression (**Fig 3A and 3B**, respectively). We next examined whether our antibiotic treatment sufficiently lowered cecum SCFAs, which included the SCFAs and intermediates in SCFA synthesis (see Methods), in our mouse model to test the relationship between the gut microbiota and FFA2 during pregnancy. Cecum samples were collected from mice prior to pregnancy (baseline) and during pregnancy (G15). As expected, we observed a significant difference between the baseline cecum SCFA concentrations in both WT and *Ffar2*^*-/-*^ mice with nearly a 50% reduction in total cecum SCFAs in response to antibiotics (**Fig 3C**). Similarly, during pregnancy, antibiotic treatment resulted in roughly a 50% reduction in SCFA concentrations in both *Ffar2*^*-/-*^ and WT mice (**Fig 3D**). These data confirm that broad range antibiotic treatment significantly reduces cecum SCFA concentrations.

**Fig 4:**
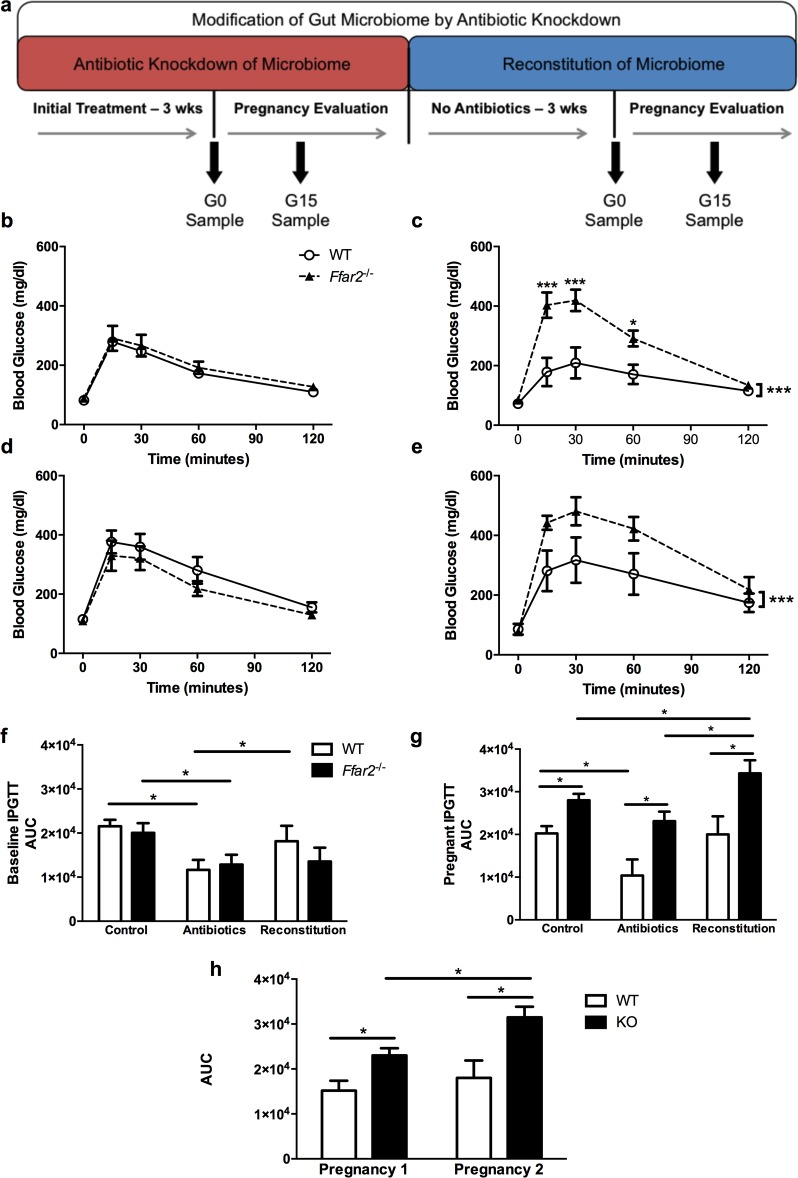
Effects of antibiotic treatment on gestational glucose tolerance before and during pregnancy. (**a**) Timeline of antibiotic treatment, pregnancy and reconstitution of the gut microbiota of female mice, where the control group was WT (*Ffar2*^+/+^) mice and the experimental group was *Ffar2*^-/-^ mice. (**b-c**) Plasma glucose concentrations during an IPGTT in antibiotic-treated mice at G0 (**b**) and at G15 (**c**). (**d-e**) Plasma glucose concentrations during an IPGTT in mice after gut microbiome reconstitution at G0 (**d**) and G15 (**e**). (**f-g**) Comparison of AUC values for plasma glucose concentrations during an IPGTT administered to control, antibiotic-treated and gut microbiota reconstituted WT and *Ffar2*^-/-^ mice prior to pregnancy (**f**) and at G15 (**g**). (**h**) Area under the curve (AUC) values for plasma glucose concentrations during an IPGTT in WT and *Ffar2*^-/-^ mice on G15 of their first and second pregnancy. WT, circles and white bars; *Ffar2*^-/-^, triangles and black bars. Data in (**b-e**) were compared by 2-way ANOVA with Bonferroni post-hoc analyses. Data in (**f-h**) were compared by Student’s t-test. (*, p<0.05; **, p<0.01; ***p<0.001), n = 7–16, mice/group.

To study the impact of the gut microbiota on glucose tolerance, we knocked down the gut microbiota and cecal SCFA levels in *Ffar2*^-/-^ and WT mice with antibiotics, and assessed FFA2-dependent glucose tolerance before pregnancy and at G15, and subsequently reconstituted the gut microbiota to reverse any antibiotic effect (**Fig 4A**). As we reported before [11], *Ffar2*^-/-^ and WT mice have similar glucose tolerance at baseline, but at G15, *Ffar2*^-/-^ mice exhibit impaired glucose tolerance (**Fig 4F and 4G**). We hypothesized that during pregnancy depletion of gut microbiota with antibiotics would impair glucose tolerance in WT mice, as reduced SCFA production would result in less SCFA-FFA2 signaling. On antibiotics, prior to pregnancy, *Ffar2*^-/-^ and WT mice responded similarly to an intraperitoneal glucose challenge (**Fig 4B and 4F**); however, antibiotic treatment improved glucose tolerance compared to mice not treated with antibiotics (see **Fig 4F**) in agreement with a previous study [17]. During pregnancy, treatment with antibiotics resulted in statistically lower plasma glucose levels during an IPGTT in WT mice at multiple time points relative to *Ffar2*^-/-^ mice (**Fig 4C and 4G**). Overall, pregnant *Ffar2*^-/-^ mice had significantly impaired glucose tolerance relative to the pregnant WT mice on antibiotics (see **Fig 4G**). These data suggest that antibiotics improved glucose tolerance independent of FFA2 expression before pregnancy, and in the WT mice at G15, but this effect was absent in pregnant *Ffar2*^-/-^ mice at G15.

Following the antibiotic treatment, we reconstituted the gut microbiota of these mice before they were mated a second time to reassess their glucose tolerance. Prior to pregnancy following the gut microbiota reconstitution, the glucose tolerance of WT and *Ffar2*^-/-^ mice were similar (**Fig 4D and 4F**). Interestingly, antibiotic treatment promoted nearly a 50% improvement in the glucose tolerance of WT and *Ffar2*^-/-^ mice before pregnancy, which almost completely disappeared after gut microbiota reconstitution in WT mice (**Fig 4D and 4F**). However, the gut microbiota reconstitution did not completely reverse the improved glucose tolerance of *Ffar2*^-/-^ mice (**Fig 4D and 4F**). During pregnancy (**Fig 4E**), impaired glucose tolerance in *Ffar2*^-/-^ mice following gut microbiota reconstitution once again occurred and was significantly impaired glucose tolerance as compared to pregnant WT mice following gut microbiota reconstitution. Interestingly, the glucose tolerance of these *Ffar2*^-/-^ mice was worse than the antibiotic-treated and control *Ffar2*^-/-^ mice during pregnancy (see **Fig 4G**).

This later observation led us to examine if multiple pregnancies in *Ffar2*^-/-^ mice leads to a worsening of glucose tolerance. Using a separate cohort of mice in serial pregnancies, we observed that the glucose tolerance of *Ffar2*^-/-^ mice did worsen in the second pregnancy (see **Fig 4H**). Together, these data suggests that 1) under non-pregnant conditions, antibiotic treatment promotes FFA2 receptor-independent improvement in glucose tolerance, 2) FFA2 contribution to gestational glucose tolerance in WT mice is not disrupted by high dose antibiotics, and 3) the impairment in glucose tolerance in *Ffar2*^-/-^ mice becomes further impaired with subsequent pregnancies.

**Fig 5:**
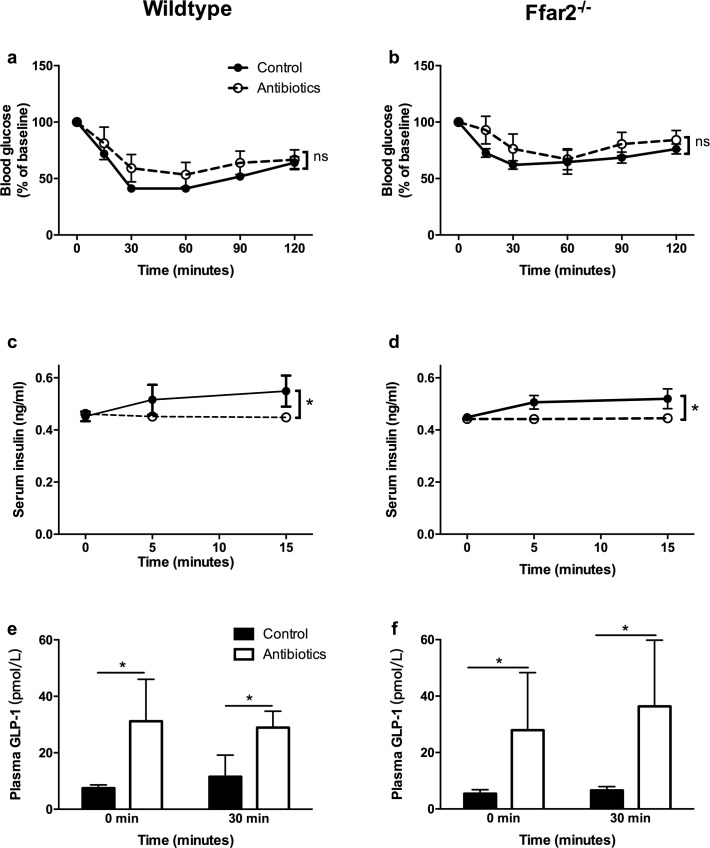
Antibiotic treatment substantially alters GLP-1 secretion independent of the mouse genotype. Insulin sensitivity, as measured by the insulin tolerance test in control female WT (**a**) and female *Ffar2*^-/-^ mice (**b**), treated with antibiotics (open circles) or untreated (filled circles), where the y axis shows the relative glucose level at each time point as compared to the glucose level at 0 min. (**c-d**) The serum insulin response to a glucose challenge in antibiotic treated mice (open symbols) compared with control mice (filled symbols) in both WT mice (**c**) and *Ffar2*^-/-^ mice (**d**). Plasma GLP-1 levels in antibiotic treated mice (white bars) compared to control mice (black bars) at 0 min and 30 min for the WT (**e**) and *Ffar2*^-/-^ mice (**f**). Data are represented as mean ± SEM. Data in (**a-d**) were compared by 2-way ANOVA with Bonferroni post-hoc analyses. Data in (**e, f**) were compared by Student’s t-test. (*, p<0.05; **, p<0.01; ***p<0.001), n = 5–8 mice/group.

To further investigate the overall improvement of glucose tolerance while on antibiotics, an effect which was independent of pregnancy, we explored if insulin tolerance was influenced in these mice (non-pregnant), where surprisingly, no difference between non-antibiotic treated WT mice or *Ffar2*^-/-^ mice compared to antibiotic treated mice was observed (**Fig 5A and 5B**). As insulin tolerance tests lack overall sensitivity, we measured insulin secretion during the IPGTT at 0, 5 and 15 mins. For WT mice and *Ffar2*^-/-^ mice (**Fig 5C and 5D,** respectively), lower insulin levels were observed while on antibiotics as compared to non-antibiotic treated, which is consistent with the overall improved glucose tolerance. As antibiotics disrupt SCFA levels through altering the gut microbiota which could possibly impaired GLP-1 secretion through a FFA2 dependent mechanism [9], we investigated if GLP-1 levels in these mice were altered at 0 min and 30 min post-glucose IPGTT. As seen in (**Fig 5E and 5F**), GLP-1 levels were profoundly elevated in antibiotic-treated mice, regardless of genotype, consistent with a previous report [20], where this elevation is possibly a major contributor to the improved glucose tolerance occurring in the antibiotic-treated mice.

### Antibiotic-mediated disruption of the gut microbiota elevates total plasma SCFAs and unequally influences individual SCFAs

**Fig 6:**
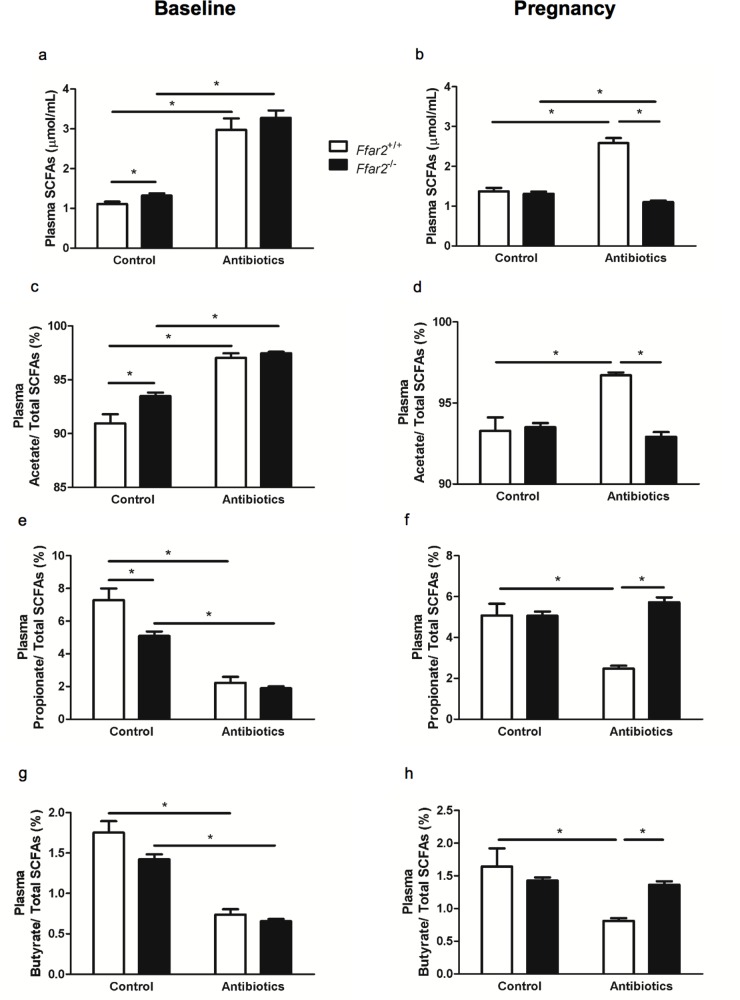
Antibiotics alter the relative abundance of individual SCFAs in circulation during pregnancy. Total plasma SCFA levels which includes acetate, propionate, and butyrate measured in WT and *Ffar2*^-/-^ mice at G0 **(a)** and G15 **(b)** under control vs antibiotic-treated conditions. (**b-h**) Relative abundance of individual SCFAs (acetate, **c-d**; propionate, **e-f**; and butyrate, **g-h**) in WT and *Ffar2*^-/-^ mice at G0 (**c, e** and **g**) and G15 (**d, f** and **h**) under control vs antibiotic-treated conditions. WT, white bars; *Ffar2*^-/-^, black bars. Data are represented as mean ± SEM n = 6–15, and were analyzed by Student’s t-test (*p ≤ 0.05).

Antibiotic suppression is a commonly used approach to disrupt the gut microbiota in order to assess the relationship between the gut microbiota and aspects of metabolism. As we anticipated, disruption of gut microbiota by antibiotic suppression led to lower cecal metabolites, SCFAs and intermediates in SCFA synthesis (succinate and lactate), in the cecum (see **Fig 3**). We expected this would translate to lower SCFA levels in circulation, where the effect of SCFAs on FFA2 function is occurring. To verify this, we next evaluated systemic SCFAs by measuring SCFA levels in plasma from blood prior to and during pregnancy in mice treated with antibiotics compared to untreated (control) mice. Unlike baseline cecum metabolites, plasma SCFA concentrations were higher in both WT and *Ffar2*^*-/-*^ mice in response to antibiotic treatment as compared to the control (**Fig 6A**). Also, during pregnancy while on antibiotics, plasma SCFA levels were elevated in WT mice, but not *Ffar2*^*-/-*^ mice (**Fig 6B**). Importantly, these data suggest that while antibiotic treatment reduces gut SCFA concentrations, this does not translate to lower SCFAs in the blood.

In β cells, the FFA2 receptor is primarily stimulated by circulating SCFAs, in particular acetate and, to a lesser degree, propionate and butyrate [21,22]. Thus, we next investigated whether antibiotics increased all blood SCFAs or if a restructuring of the relative abundance of individual SCFAs occurred. Interestingly, after antibiotic treatment, the percent of circulating acetate increased and became similar between both *Ffar2*^*-/-*^ and WT mice (**Fig 6C**). During pregnancy while on antibiotics, the percent of circulating acetate in WT mice remained elevated, but was not increased in *Ffar2*^*-/-*^ mice (**Fig 6D**). As opposed to acetate, the relative abundance of propionate in circulation decreased in *Ffar2*^*-/-*^ and WT mice at baseline on antibiotics (**Fig 6E**). During pregnancy, propionate remained decreased in WT mice on antibiotics, which did not occur with antibiotic-treated *Ffar2*^*-/-*^ mice (**Fig 6F**). The same pattern seen with propionate occurred with butyrate before and during pregnancy while on antibiotics (**Fig 6G and 6H**). Together these data indicate that broad-spectrum antibiotics increase total SCFAs in the blood, but differentially impact the concentrations of individual SCFAs, and these changes are also influenced by pregnancy.

## Discussion

It is well known that gestational diabetes can lead to adverse metabolic outcomes for both the mother and the child [12,13,14]. Our previous report established that FFA2 is involved in maintaining gestational glucose homeostasis [11]. Kahraman *et al*. recently showed that maternal insulin resistance and transient hyperglycemia lead to hyperglycemia in male and female offspring that becomes prominent (nearly 0.5-fold increase relative to wildtype controls) just 10 days after birth [23]. Because of the known influence of elevated glucose levels on fetal health, we examined if the impaired glucose tolerance in our female *Ffar2*^-/-^ mice during pregnancy influenced the metabolic fitness of their offspring. However, no overt metabolic effects on the offspring occurred. Unlike the liver specific insulin receptor knockout (LIRKO) mouse used in the previous study [23], our *Ffar2*^-/-^ mice do not exhibit elevated plasma insulin concentrations (see [11]) suggesting possibly that maternal hyperinsulinemia may be driving the early-age metabolic impacts in the offspring. It is also possible that the degree of maternal hyperglycemia in *Ffar2*^-/-^ mice is not sufficient to induce acute metabolic effects on the offspring [23], as the degree of impairment in the LIRKO mice is more severe than that of the *Ffar2*^-/-^ mice during pregnancy. As apparent, this model establishes that FFA2 contributes to gestational glucose homeostasis, but the metabolic health of the offspring over the first 6–7 weeks of life is not influenced.

An important question is why we do not see altered weight gain with our *Ffar2*^-/-^ mice as compared to WT mice, as reported by others [24]. In their report, genetic deletion or overexpression of FFA2 in adipose tissue resulted in either obese mice on normal diets or lean mice on high fat diets, relative to WT mice. While this study suggests a role of FFA2 in fat accumulation, multiple other groups [4, 5, 25] have not observed a role of FFA2 in adiposity (see [26] for complete discussion). The discrepancies in these studies are likely due to the complexity of FFA2 signaling, the existence of other SCFA receptors, and the unclear influence of the gut microbiota, which is known to be unique between animal facilities.

In this study, long-term treatment with antibiotics resulted in a sizeable reduction in cecum SCFAs in non-pregnant and pregnant mice, and this was likely from a reduced gut bacterial content, as observed here. Our data also shows that antibiotic treatment promotes FFA2-independent improvement in glucose tolerance under non-pregnant conditions as previously described [17], which was reversed upon reconstitution of the gut microbiota. This effect of improved glucose tolerance has been observed by others with gut microbiota knockdown through antibiotics and also under germ free conditions [17,27]; however, it is not clear how this is occurring. While our data with the antibiotic treated mice does not indicate improved insulin tolerance, possibly due to the lack of sensitivity of this test, we observed profoundly elevated GLP-1 levels regardless of genotype while the mice were on antibiotics, consistent with a previous report [20]. Moreover, with the antibiotic treated mice, insulin levels were lower in our report and the previous one [20], indicating improved insulin resistance. As GLP-1 is known to improve insulin sensitivity, this may be an important reason why antibiotic-treated mice and possibly germ-free mice have improved glucose tolerance.

While we expected that antibiotic ablation of the gut microbiota and the corresponding reduction in cecum metabolites would translate into reduced SCFAs in the plasma, we observed the opposite, that antibiotics led to increased total plasma SCFA concentrations and relative plasma acetate levels in WT mice, indicating that antibiotic treatment, at least the protocol used in our study, is not sufficient to suppress circulating blood SCFAs. In addition to bacterial fermentation, the gut microbiota also determines intestinal architecture and modulates intestinal barrier function by maintaining a 50μm thick mucus layer and intestinal epithelial cell junctions, which can limit the absorption of microbial and luminal contents [28–32]. A recent study reported that antibiotic perturbation of the microbiota leads to a reduction in the thickness of the colon wall and protective mucus layer [33], providing a potential mechanism through which increased transport of SCFAs from the lumen of the gut may be occurring and enter the surrounding vasculature. Interestingly, metronidazole, one of the antibiotics used in our study, have been shown to alter both microbiota and goblet cell function resulting in lower *mucin-2* expression and subsequent thinning of the mucus layer [33]. Our data raised the important concern that gut microbiota ablation with antibiotics may not always be sufficient to lower blood SCFA levels. Additionally, this increase in plasma levels of SCFAs was not observed in pregnant *Ffar2*^-/-^ mice on antibiotics, which may be for a multitude of reasons. For example, SCFA transport is a highly regulated process within the intestinal epithelium [34], where nutrient sensing receptors such as FFA2 may contribute to SCFA transport. Additionally, the gut microbiota of the *Ffar2*^-/-^ mice could have been influenced differently during pregnancy than the WT mice, and/or the influence of the antibiotics on the gut microbiota during pregnancy may have been unique between the mouse genotypes. Moreover, energy intake and/or demand could also be influenced in these pregnant *Ffar2*^-/-^ mice, through unclear mechanisms. Future studies will require gnotobiotic mouse models to definitively determine the function of FFA2 in intestinal SCFA transport.

In our study, the altered plasma SCFA profile was associated with improved baseline glucose tolerance. We expect, that during pregnancy, the effect of FFA2 signaling in the β cell as a result of increased plasma acetate levels led to improved gestational glucose tolerance in WT mice while on antibiotics. Interestingly, *Ffar2*^-/-^ mice did not have elevated blood SCFA levels during pregnancy (and WT mice did have elevated SCFA levels), making it complicated to interpret our results on the role of SCFAs/FFA2 signaling during pregnancy in *Ffar2*^-/-^ mice. To address this in a different manner, we adapted an approach from Chen *et al*. [35], where we supplemented the drinking water of WT and *Ffar2*^-/-^ mice with 1% sodium acetate to directly test the effect of increased acetate levels on FFA2-dependent β cell function. Unfortunately, oral acetate supplementation alone was not sufficient to elevate plasma acetate concentrations in this model to test this question (data not shown). To help understand how SCFA levels influence different aspects of glucose metabolism in an FFA2 dependent and independent manner may require a venous infusion approach to regulate blood SCFA levels in the future.

The results of this study raise two major questions: 1) what changes are occurring in the gut microbiota composition and its SCFA production abilities from antibiotic treatment, and 2) do antibiotics impact the factors regulating the transport of SCFAs from the cecum into the blood? To answer this first question, the post-antibiotic treated community of gut microbes must be definitively established, along with SCFA production levels from each treatment. These data will help to validate antibiotic approaches to modulate these factors. Moreover, these data will be helpful in understanding how the gut microbiota ultimately drives circulating SCFA levels. Addressing the second question will rely on the interrogation of the mechanisms that regulate cecum SCFA transport, including thickness of the protective mucus layer, colonic absorption rates, etc. One likely possibility is that antibiotic treatment leads to thinning of the gut’s epithelial lining, as shown in a similar study [33], allowing for increased absorption of gut-derived metabolites into circulation. Overall, our study provides new directions for additional investigation to understand the impact of antibiotics on circulating gut-derived metabolite concentrations and SCFA receptor signaling.

In this study, we also determined that subsequent pregnancies lead to further worsening of glucose tolerance impairment in the *Ffar2*^-/-^ mice during pregnancy. This result can also occur in humans where GDM patients exhibit a higher rate of developing GDM in subsequent pregnancies and also T2D later in life [36]. Our data also demonstrate that antibiotic perturbation of mouse gut microbiota leads to improved glucose tolerance before and during pregnancy possibly from increased acetate concentrations in the circulation. Insights into the mechanism through which plasma acetate levels are elevated during antibiotic treatment could provide clues for future strategies to improve glucose tolerance. In sum, these data have added to our understanding of the role of FFA2 in gestational regulation of glucose and provide insight into needed further investigation.

## Supporting Information

S1 Fig This file contains the data for each figure. (XLSX)
